# Pentoxazone (Second Edition) (Pesticides)

**DOI:** 10.14252/foodsafetyfscj.D-25-00014

**Published:** 2025-06-27

**Authors:** Food Safety Commission of Japan

## Abstract

Food Safety Commission of Japan (FSCJ) conducted a risk assessment of pentoxazone (CAS No. 110956-75-7), an oxazolidine herbicide, based on submitted documents. A request for reevaluation was made under the Agricultural Chemical Regulation Act. Additional information was submitted by the Ministry of Agriculture, Forestry and Fisheries, which included data on fate in livestock (goats and chickens), genotoxicity, and related published scientific literatures. Major adverse effects of pentoxazone were observed in the liver (hepatocellular hypertrophy) and urinary bladder (proliferative lesions including mucosal epithelial hyperplasia). Adverse effects were observed on neither fertility, teratogenicity, nor biologically significant genotoxicity. The lowest no-observed-adverse-effect level (NOAEL) obtained from these studies was 23.1 mg/kg bw per day in the one-year chronic toxicity study in dogs. FSCJ specified an acceptable daily intake (ADI) of 0.23 mg/kg bw per day by applying a safety factor of 100 to this NOAEL.

## Conclusion in Brief

Food Safety Commission of Japan (FSCJ) conducted a risk assessment of pentoxazone (CAS No. 110956-75-7), an oxazolidine herbicide, based on submitted documents. A request for reevaluation was made under the Agricultural Chemical Regulation Act. Additional information was submitted by the Ministry of Agriculture, Forestry and Fisheries, which included data on fate in livestock (goats and chickens), genotoxicity, and related published scientific literatures.

The data used in the assessment include fate in plants (paddy rice), residues in crops, fate in livestock (goats and chickens), fate in animals (rats), subacute toxicity (rats, mice, and dogs), chronic toxicity (dogs), combined chronic toxicity/carcinogenicity (rats), carcinogenicity (mice), toxicity (mice), two-generation reproductive toxicity (rats), developmental toxicity (rats and rabbits) and genotoxicity.

Major adverse effects of pentoxazone were observed in the liver (hepatocellular hypertrophy) and urinary bladder (proliferative lesions including mucosal epithelial hyperplasia). Adverse effects were observed on neither fertility, teratogenicity, nor biologically significant genotoxicity. An increase in diffuse epithelial hyperplasia of the urinary bladder was observed in rats of both sexes in the two-year combined chronic toxicity/carcinogenicity study. Furthermore, an increased incidence of transitional cell papilloma in the urinary bladder was observed in female rats. The mode of action was, however, considered to be non-genotoxic, and it was deemed possible to establish a threshold for evaluation.

Based on these results, pentoxazone (parent compound only) was identified as the relevant substance for the residue definition for dietary risk assessment in agricultural and fishery products.

The lowest no-observed-adverse-effect level (NOAEL) obtained from these studies was 23.1 mg/kg bw per day in the one-year chronic toxicity study in dogs. FSCJ specified an acceptable daily intake (ADI) of 0.23 mg/kg bw per day by applying a safety factor of 100 to this NOAEL.

Since there was no adverse effect likely to be elicited by a single oral administration of pentoxazone, it is thus considered unnecessary to specify an acute reference dose (ARfD). 

**Table 1.** *Levels relevant to toxicological evaluation of pentoxazone*

Species	Study	Dose (mg/kg bw per day)	NOAEL (mg/kg bw per day)	LOAEL (mg/kg bw per day)	Critical endpoints^1^^)^
Rat	90-day subacute toxicity study	0, 80, 400, 2 000, 10 000 ppm	M: 117 F: 26.1	M: 606 F: 129	M/F: Bile duct hyperplasia, etc.
		M: 0, 4.65, 23.6, 117, 606 F: 0, 5.24, 26.1, 129, 664			
	Two-year combined chronic toxicity/ carcinogenicity study	0, 200, 1 000, 5 000 ppm	M: 35.2 F: 43.8	M: 181 F: 225	M/F: Diffuse epithelial hyperplasia of the urinary bladder, (F: Increased incidence of transitional cell papilloma in the urinary bladder)
		M: 0, 6.92, 35.2, 181 F: 0, 8.74, 43.8, 225			
	Two-generation reproductive toxicity study	0, 50, 1 000, 10 000 ppm	Parents and offspring PM: 71.2 PF: 84.5 F_1_M: 85.5 F_1_F: 98.6	Parents and offspring PM: 716 PF: 821 F_1_M: 858 F_1_F: 986	Parents M/F: Increased relative weights of the liver, etc. Offspring M/F: Low body weights postnatal day 21 (No effect on fertility is observed)
		PM: 0, 3.57, 71.2, 716 PF: 0, 4.07, 84.5, 821 F_1_M: 0, 4.14, 85.5, 858 F_1_F: 0, 4.81, 98.6, 986			
	Developmental toxicity study	0, 40, 200, 1 000	Dams: 1 000 Fetuses: 1 000	Dams: - Fetuses: -	Dams: No toxicity Fetuses: No toxicity (No teratogenicity observed)
Mouse	90-day subacute toxicity study	0, 80, 400, 2 000, 10 000 ppm	M: 251 F: 54.3	M: 1 240 F: 271	M: Increase in epithelial hyperplasia of the urinary bladder, etc. F: Eosinophilic body deposition in the bladder epithelium
		M: 0, 9.79, 48.0, 251, 1 240 F: 0, 10.9, 54.3, 271, 1 430			
	18-month carcinogenicity study	0, 80, 400, 2 000 ppm	M: 203 F: 191	M: - F: -	M/F: No toxicity (No carcinogenicity is observed)
		M: 0, 7.88, 41.4, 203 F: 0, 7.59, 37.1, 191			
Rabbit	Developmental toxicity study	0, 100, 300, 1 000	Dams: 100 Fetuses: 100	Dams: 300 Fetuses: -	Dams: Death, abortion, premature birth, etc. Fetuses: No toxicity (No teratogenicity observed)
Dog	90-day subacute toxicity study	0, 400, 2 000, 10 000 ppm	M: 58.8 F: 64.3	M: 312 F: 318	M/F: Increase in ALP, hepatocellular hypertrophy, etc.
		M: 0, 12.3, 58.8, 312 F: 0, 13.2, 64.3, 318			
	One-year chronic toxicity study	0, 200, 1 000, 5 000 ppm	M: 23.1 F: 25.2	M: 113 F: 121	M/F: Increase in ALP, hepatocellular hypertrophy, etc.
		M: 0, 4.50, 23.1, 113 F: 0, 4.76, 25.2, 121			
ADI			NOAEL: 23.1 SF: 100 ADI: 0.23		
The critical study for setting ADI			One-year chronic toxicity test (dog)		

ALP, Alkaline phosphatase; ADI, Acceptable daily intake; NOAEL, No-observed-adverse-effect level;

LOAEL, Lowest-observed-adverse-effect level; SF, Safety factor

^1)^ The adverse effect observed at LOAEL

-: LOAEL could not be specified.
